# Methodologies Used in Remote Sensing Data Analysis and Remote Sensors for Precision Agriculture

**DOI:** 10.3390/s22207898

**Published:** 2022-10-17

**Authors:** Sigfredo Fuentes, Jiyul Chang

**Affiliations:** 1 Digital Agriculture Food and Wine, School of Agriculture and Food, Faculty of Veterinary and Agricultural Science, The University of Melbourne, Parkville, VIC 3010, Australia; 2 Department of Agronomy, Horticulture & Plant Science, South Dakota State University, Brookings, SD 57007, USA

When adopting remote sensing techniques in precision agriculture, there are two main areas to consider: data acquisition and data analysis methodologies. Imagery and remote sensor data collected using different platforms provide a variety of information volumes and formats. For example, recent research in precision agriculture has used multispectral images from different platforms, such as satellites, airborne, and, most recently, drones. These images have been used for various analyses, from the detection of pests and diseases, growth and water status of crops, to yield estimations. However, accurately detecting specific biotic or abiotic stresses requires a narrow range of spectral information to be analyzed for each application. In data analysis, the volume and complexity of data formats obtained using the latest technologies in remote sensing (e.g., a cube of data for hyperspectral imagery) demands complex data processing systems and data analysis using multiple inputs to estimate specific categorical or numerical targets. New and emerging methodologies within artificial intelligence, such as machine learning and deep learning, have enabled us to deal with these increasing data volumes and complex analyses.

This Special Issue (SI) mainly focused on (i) advanced methodologies for remotely sensed data collected by different types of sensors and platforms for precision agriculture and (ii) the implementation of various sensors for specific targets in precision agriculture. High-quality research was published in this SI from researchers from various countries, including China, the USA, Slovenia, Spain, Germany, Brazil, Australia, and Singapore. The SI’s studies have been ordered following the application within the soil–plant–atmosphere continuum starting with the soil salinity precision monitoring using unmanned aerial vehicles (UAV) and multispectral imagery [1]; the evaluation of optical sensors for the diagnosis of nitrogen content for wheat plants [2]; the detection of root-knot nematode infestation in potato plants using hyperspectral imagery [3]; detection of powdery mildew using hyperspectral, thermal, and RGB imagery [4]; leaf area index estimations for wheat using hyperspectral reflectance data [5]; vineyard canopy characteristics and vigor assessment using UAV and satellite imagery [6]; estimation of crop vegetation parameters using satellite and UAV spectral remote sensing [7]; above-ground biomass estimation of oat plants using UAV remote sensing and machine learning [8]; wheat lodging estimation using multispectral UAV imagery and deep learning [9]; yield estimation for guinea grass using UAV remote sensing [10]; and wheat yield prediction from satellite imagery, meteorological data, and machine learning modeling [11].

Different sensor technologies, such as SPAD, Dualex 4, and RapidSCAN, were implemented to assess the accuracy of estimating nitrogen levels in winter wheat, with Dualex 4 being the sensor with the best performance [2].

Different machine and deep learning analytical methods were employed to analyze imagery and the numerical data from various research studies. For soils, among the methodologies used were partial least square (PLS) back propagation neural networks (BPNN), support vector machines (SVM), and random forests (RF) to construct retrieval models to estimate soil salinity using regression models [1]. The latter was the most accurate method resulting in determination coefficients of R^2^ = 0.724 for the modeling stage and 0.745 for validation. For roots and disease estimation, hyperspectral imagery for disease detection on potato tubers (diseased and non-diseased), and machine learning modeling using SVM classifiers plus dimensionality reduction methods with accuracies over 60% [3]. Other multisource vegetation indices extracted from hyperspectral, thermal, and RGB imaging have been used coupled with RF and SVM regression algorithms to target a powdery mildew index on wheat. The former machine learning methodology resulted in higher and more stable performances and R^2^ > 0.86 [4]. 

In terms of canopy architecture, hyperspectral reflectance data from winter wheat was used as inputs for a combination of algorithms at different phenological stages to estimate LAI as targets. The best performance was obtained in flowering and filling stages with 0.87 < R^2^ < 0.71 for modeling and 0.84 < R^2^ < 0.77 for validation, respectively [5]. Other canopy-related parameters for vineyards, such as the normalized differential vegetation index (NDVI) obtained from UAV and satellite multispectral data using simple linear regression from individual plants and clusters of plants according to the spatial footprint of imagery. The NDVI was then related to the tree row volume resulting in moderate R^2^ for vigor estimation [6]. Other multispectral/hyperspectral parameters from satellite–UAV data comparisons were performed to estimate crop vegetation parameters, such as LAI, leaf chlorophyll concentration, and canopy water content, with no clear superiority for either remote-sensed data on the estimations [2]. For above-ground biomass estimation of oats, UAV-based remote sensing multispectral imagery and derived vegetation indices (VIs) were coupled with PLS, SVM, and artificial neural networks (ANN) and RF algorithms. These studies’ results showed various low to moderate accuracies in predicting above-ground biomass [8]. The highest accuracy was obtained by combining RGB + digital surface model (DSM) with 89% [9]. Deep learning based on convolutional neural networks (CNN) with different architectures to analyze RGB from a UAV platform was used to estimate dry matter yield for guinea grass resulting in correlation coefficients of 0.79 < R < 0.62 [10]. Furthermore, RF algorithms were also used for wheat yield prediction based on satellite-based NDVI combined with meteorological data in Australia, resulting in 0.89 < R2 < 0.42 for different locations [11].

After climatic anomalies, plants can suffer from lodging, such as wheat, and the damage estimation can be helpful in decision-making. Multispectral imagery from a UAV platform was used to estimate lodging in wheat coupled with a lightweight network model method based on RGB-DSM with 89% accuracy in the lodging estimation.

It has been shown in this SI that remote sensing coupled with artificial intelligence and machine learning are powerful tools to estimate parameters from soil salinity, plant biotic and abiotic stresses/damage, canopy architecture characteristics, and yield estimation.

## Data Availability

Not applicable.

## References

[1] Yu X., Chang C., Song J., Zhuge Y., Wang A. (2022). Precise Monitoring of Soil Salinity in China’s Yellow River Delta Using UAV-Borne Multispectral Imagery and a Soil Salinity Retrieval Index. Sensors.

[2] Jiang J., Wang C., Wang H., Fu Z., Cao Q., Tian Y., Zhu Y., Cao W., Liu X. (2021). Evaluation of Three Portable Optical Sensors for Non-Destructive Diagnosis of Nitrogen Status in Winter Wheat. Sensors.

[3] Lapajne J., Knapič M., Žibrat U. (2022). Comparison of Selected Dimensionality Reduction Methods for Detection of Root-Knot Nematode Infestations in Potato Tubers Using Hyperspectral Imaging. Sensors.

[4] Feng Z., Song L., Duan J., He L., Zhang Y., Wei Y., Feng W. (2022). Monitoring Wheat Powdery Mildew Based on Hyperspectral, Thermal Infrared, and RGB Image Data Fusion. Sensors.

[5] Li C., Wang Y., Ma C., Ding F., Li Y., Chen W., Li J., Xiao Z. (2021). Hyperspectral Estimation of Winter Wheat Leaf Area Index Based on Continuous Wavelet Transform and Fractional Order Differentiation. Sensors.

[6] Campos J., García-Ruíz F., Gil E. (2021). Assessment of Vineyard Canopy Characteristics from Vigour Maps Obtained Using UAV and Satellite Imagery. Sensors.

[7] Wijesingha J., Dayananda S., Wachendorf M., Astor T. (2021). Comparison of Spaceborne and UAV-Borne Remote Sensing Spectral Data for Estimating Monsoon Crop Vegetation Parameters. Sensors.

[8] Sharma P., Leigh L., Chang J., Maimaitijiang M., Caffé M. (2022). Above-Ground Biomass Estimation in Oats Using UAV Remote Sensing and Machine Learning. Sensors.

[9] Yang B., Zhu Y., Zhou S. (2021). Accurate Wheat Lodging Extraction from Multi-Channel UAV Images Using a Lightweight Network Model. Sensors.

[10] De Oliveira G.S., Marcato Junior J., Polidoro C., Osco L.P., Siqueira H., Rodrigues L., Jank L., Barrios S., Valle C., Simeão R. (2021). Convolutional Neural Networks to Estimate Dry Matter Yield in a Guineagrass Breeding Program Using UAV Remote Sensing. Sensors.

[11] Pang A., Chang M.W.L., Chen Y. (2022). Evaluation of Random Forests (RF) for Regional and Local-Scale Wheat Yield Prediction in Southeast Australia. Sensors.

